# Systematic Review of Intraoperative Radiotherapy (IORT) in Head and Neck Oncology: Past, Present, and Future Perspectives

**DOI:** 10.3390/cancers17132124

**Published:** 2025-06-24

**Authors:** Laurence Pincet, Aurelie Fanchette, Jolanda Elmers, Jean Bourhis, Karma Lambercy, Edouard Romano

**Affiliations:** 1 ENT, Head and Neck Department, CHUV, Rue du Bugnon 46, 1010 Lausanne, Switzerland; laurence.pincet@chuv.ch (L.P.); karma.lambercy@chuv.ch (K.L.); 2 Pole Formation, Recherches–Soutien aux Revus Systématiques, Bibliothèque Universitaire de Médecine, Chemin des Falaises 2, 1005 Lausanne, Switzerland; jolanda.elmers@chuv.ch; 3 Radiation Oncology Department, CHUV, Rue du Bugnon 46, 1010 Lausanne, Switzerland; jean.bourhis@chuv.ch (J.B.); edouard.romano@chuv.ch (E.R.)

**Keywords:** intraoperative radiotherapy (IORT), head and neck cancer, local control, recurrence, radiation therapy, complications, advanced tumors, oncologic surgery

## Abstract

**Background/Objectives:** Intraoperative radiotherapy (IORT) has been used for decades to improve local control in advanced and recurrent head and neck cancers by delivering a concentrated dose of radiation directly to the tumor bed during surgery. Despite its potential advantages, IORT remains controversial due to its high complication rates and logistical complexity. This systematic review aims to assess the current evidence on IORT in head and neck oncology, evaluating its indications, effectiveness, and limitations. **Methods:** A systematic literature review was conducted in accordance with Cochrane systematic review method and reported following PRISMA guidelines. Databases, including Medline, Embase, Cochrane, and Web of Science, were searched for studies evaluating IORT in head and neck cancer. Outcomes of interest included local control rates, overall survival, complications, and treatment logistics. Data were weighted based on patient numbers, and statistical analyses included weighted means and comparative tests. **Results:** Included in this review are 47 studies that included 2330 patients. The studies were highly heterogeneous, limiting definitive conclusions. IORT was mainly used in stage III/IV or recurrent tumors, with an average dose of 14.7 Gy (range: 1–40 Gy). The five-year local control was 47% to 82%. Complication rates included wound necrosis (22,3%), fistulas 34 (17.8%), and carotid blow-out (14.5%). Logistical constraints remain a major limitation. **Conclusions:** The heterogeneous data and logistic challenges of IORT in head and neck oncology have limited its use, and the benefits remain uncertain.

## Simple Summary

Intraoperative radiotherapy (IORT) is a technique that delivers a high dose of radiation directly to the tumor bed during surgery, aiming to improve local control while sparing surrounding healthy tissues. It has been used primarily in advanced or recurrent head and neck cancers, especially in patients previously treated with external beam radiotherapy. While promising, the evidence remains limited to mostly retrospective, single-institution studies with heterogeneous methodologies and outcomes. Complication rates can be high, and logistical challenges such as equipment availability and surgical coordination limit its broader use. This review examines current indications, outcomes, and practical limitations of IORT.

## 1. Introduction

Intraoperative radiotherapy (IORT) is a technique in which a concentrated dose of radiation is delivered directly to a tumor site during surgery. This approach allows for the precise targeting of the tumor while minimizing exposure to surrounding healthy tissues [1]. Intraoperative radiation therapy (IORT) is the delivery of irradiation to a well-delineated target volume (today, likely using a mobile linear accelerator) within an exposed surgical field that may shield specific sites (such as the carotid artery). IORT using electrons or photons has, over the past 50 years, most commonly been part of a re-irradiation regimen following salvage surgery for recurrences, often with planned additional external beam irradiation. It has shown a benefit in local control, especially when surgical margins were no worse than microscopically positive [2,3]

IORT has been widely studied and used in the treatment of early-stage breast cancer, often delivered as a single fraction during lumpectomy to replace or supplement external beam radiation therapy (EBRT) [3]. It is also applied in treating pancreatic, rectal, and other gastrointestinal cancers, as well as soft tissue sarcomas, particularly in challenging anatomical regions [4,5].

Recent reviews, such as that of Villafuerte et al. [6], have summarized outcomes across 52 studies and 2389 patients, including both IORT and brachytherapy-based interventions. In contrast, the present review focuses exclusively on external beam-based IORT to provide a more targeted analysis of its oncologic indications, outcomes, and complications. Our goal is to supplement and refine previous findings through a focused synthesis that excludes brachytherapy studies.

In head and neck cancer, IORT offers significant potential in treating these cancers, which often involve complex anatomical regions. It allows for precise high-dose radiation delivery immediately after surgical resection, improving local control and reducing recurrence risk [5,7]. This paper will comprehensively review the literature of IORT in the head and neck, discussing indications, strengths, and limitations.

## 2. Methods

To identify articles, we searched the following databases, using a combination of controlled vocabulary terms and free text terms covering head and neck cancer and intraoperative radiotherapy: Medline OVID SP, Embase.com, Cochrane Central Register of Controlled Trials (via Cochrane Library), and Web of Science Core Collection, with supplementary searches in ClinicalTrials.gov, WHO ICTRP, and Google Scholar.

**Figure 1:**
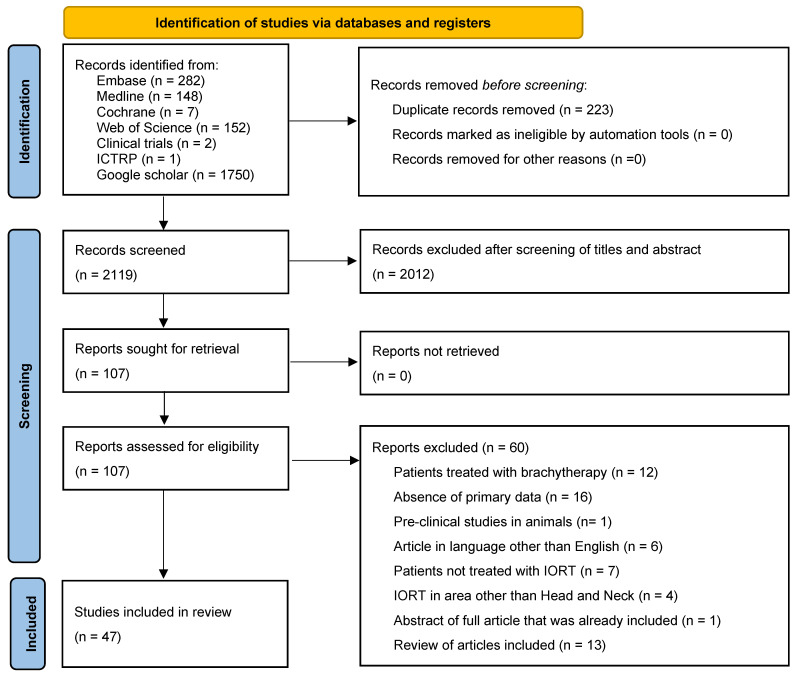
PRISMA 2020 flow diagram for new systematic reviews which include searches of databases and registers only.

**Figure 2:**
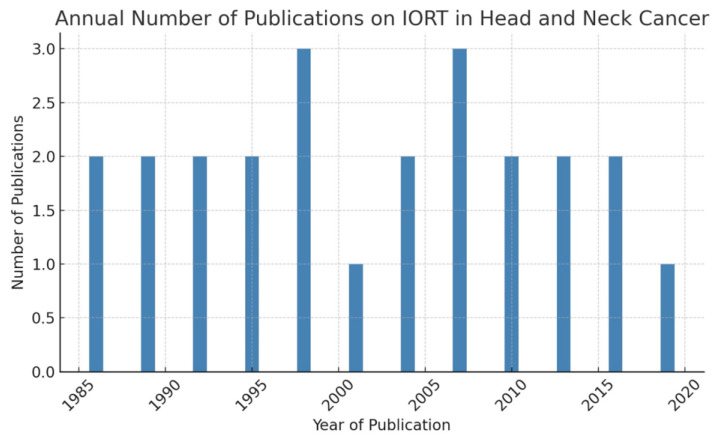
Number of IORT publications.

Forty-seven articles from 1986 to 2019 are included (Table 1, Figure 1 and Figure 2), with a total of 2330 patients and 2420 irradiation fields (WAM = 49, WM = 40, range = 1–231; WSD = 42). Eighteen articles were from Europe and two were from Asia.

When the doses delivered are indicated as a ‘range’ without details, we assumed a homogeneous distribution of patients within the given range. Statistical significance testing was performed using chi-square tests, Mann–Whitney U tests and *T*-tests.

We categorized complications into two categories comprising transient, short-to-medium term consequences or permanent, long-term consequences. We followed the Clavien–Dindo classification scheme [2] in assessing acute adverse events, and the CTCAE classification when focusing on long-term effects [4].

This systematic review is conducted based on Cochrane systematic review method and reported in accordance with the PRISMA 2020 (Preferred Reporting Items for Systematic Reviews and Meta-Analyses) guidelines. The review was not registered in PROSPERO or other registries.

PRISMA Compliance and Risk of Bias: This review was conducted in accordance with PRISMA 2020. It was not registered in PROSPERO, as the project began prior to formal registration planning. The selection of studies was conducted using the Rayyan platform (Qatar Computing Research Institute), which ensures blinding between reviewers and highlights conflicts automatically. Two independent reviewers screened all records and assessed eligibility in a blinded manner. Disagreements were resolved by a third reviewer, who made the final inclusion decision. The included studies were predominantly retrospective and exhibited moderate to high risk of bias due to non-randomized designs, heterogeneity in protocols, inconsistent outcome reporting, and limited control for confounding factors (Supplementary Materials).

## 3. Results

Our literature review included 47 articles (Table 1, Figure 1 and Figure 2) with varying numbers of patients (WAM = 49, WM = 40, range = 1–231; WSD = 42). These articles were published between 1986 and 2019; the origin of the publications was the USA for thirty-one articles (60%), Europe for eighteen (36%), and Asia for two (4%). This represents a total of 2330 patients, or 2420 irradiation fields.

### 3.1. Patients’ Characteristics (Table 2, Figure 3)

Among the 1.640 specified irradiated fields (68% of total) the distribution of irradiated sites was as follows: neck (466, 45%), oral cavity (166, 27%), parotid or submandibular glands (263, 25%), oropharynx (192, 18%), skull base (144, 14%), hypopharynx (126, 12%), larynx (73, 7%), nasopharynx (66, 6%), facial skin (43, 4%), temporal fossa (23, 2%), external or middle ear (16, 2%); and ≤1% pterygoid fossa [46], maxillary sinus [14], thyroid [36], prevertebral infiltration [5], orbit [3], and nasal fossa [2]. The authors reported data for 804 patients (35% of total):132 primary tumors (16%) and 675 recurrences (84%). Histological margins were reported in 398 patients (17% of total): 113 R0 or close margins (28%), 165 microscopic infiltration (R1, 41%), and 120 with macroscopic infiltration (R2, 30%).

Histology was reported in 60% of patients (1397): squamous cell carcinoma (SCC) in 1197 (86%), salivary gland (144 cases, 10%), thyroid [papillary, follicular, anaplastic, and undifferentiated carcinomas] (22; 1.6%); skin SCC (14; 1%), sarcomas [rhabdomyosarcoma, synovial cell sarcoma, and myxoliposarcoma] (7; 0.5%), esthesioneuroblastoma (4; 0.3%), neuroendocrine carcinoma [2], cutaneous melanoma (2 patients) [2]; and single cases of chondroma, large cell carcinoma, lymphoepithelial carcinoma, histiocytoma, and non-Hodgkin lymphoma.

Initial tumor stage was reported in 731 patients (30%): among these, 186 (25%) were stage I–II, and 545 (75%) were stage III–IV. Among 807 patients with data, 84% (675) were recurrences and 165 were primary (132).

**Table 2:** Patients’ characteristics.

Patients’ Characteristics:		
Location (Data from 1640 irradiated fields, representing 68% of the total)		
- Lymph nodes - Oral cavity - Parotid/submandibular gland - Oropharynx2 - Skull base - Hypopharynx - Larynx - Nasopharynx - Face skin - Temporal region - External or middle ear - Pterygoid fossa - Maxillary sinus - Thyroid - Prevertebral involvement - Orbit - Nasal fossa	466 279 263 192 144 126 73 66 43 23 16 11 10 8 5 3 2	45% 27% 25% 18% 14% 12% 7% 6% 4% 2% 2% 1% 1% 1% 0.5% 0.3% 0.2%
**Primary vs. Recurrence** (*Data from 804 patients, representing 35% of the total*)		
- Primary - Recurrence	132 675	16% 84%
**Margins** (*Data from 398 patients, representing 17% of the total*)		
- R0, closed margins - R1 - R2	113 165 120	28% 41% 30%
**Stage** (*Data from 731 patients, representing 31% of the total*)		
- Stage I or II - Stage III or IV	186 545	25% 75%
**Histology** (*Data from 1397 patients, representing 60% of the total*)		
- Squamous cell carcinoma - Salivary gland tumor ^1^ - Thyroid ^2^ - Skin Squamous cell carcinoma - Sarcomas ^3^ - Esthesineuroblastoma - Neuroendocrine carcinoma - Cutaneous melanoma - Chondroma - Large cell carcinoma - Lymphoepithelial carcinoma - Histiocytoma - Non-Hodgkin lymphoma	1197 144 22 14 7 4 2 2 1 1 1 1 1	85.7% 10.3% 1.6% 1.0% 0.5% 0.3% 0.1% 0.1% 0.1% 0.1% 0.1% 0.1% 0.1%

^1^ Salivary gland tumor: Adenocarcinoma, mucoepidermoid carcinoma, and adenoid cystic carcinoma. ^2^ Thyroid: Papillar, follicular, anaplastic, and undifferentiated carcinomas. ^3^ Sarcomas: Rhabdomyosarcome, synovial cell sarcoma, and myxoliposarcoma.

**Figure 3:**
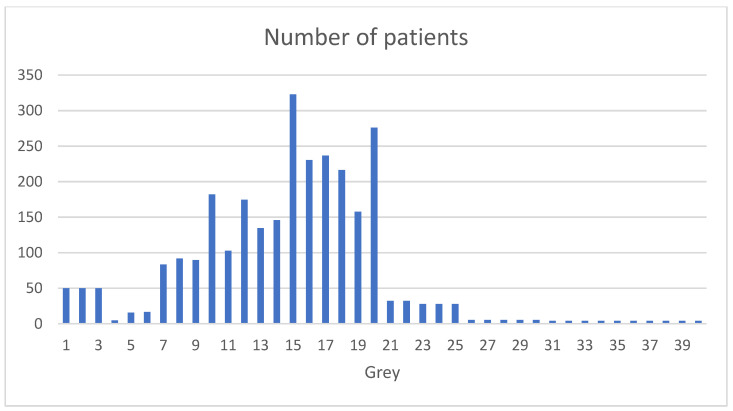
Irradiation dosage per patient.

### 3.2. Radiation Characteristics

The doses delivered varied significantly, ranging from 1 to 40 Gy in one fraction (WAM = 14.7 Gy; WSD = 5.78). The cases often described situations in which the irradiation field included critical structures such as the carotid artery, brain tissue, or nerve structures. When this was so, the dose was usually <10 Gy, but the relevant data is insufficient to conduct statistical analysis. Most surgery centers use photons. Some use electrons, ranging from 4 to 18 MeV. This results in irradiation depths from 0.5 to 8 cm. Few authors describe the procedure time. Some have described the irradiation time [5,7] (1.5–20 min), while others have described the time taken by the entire irradiation procedure, which corresponds to a 25 to 75 min surgery-interruption time [7,14,19,30,32,36].

### 3.3. Outcomes

The results are highly heterogeneous, varying depending on the number of patients, type of tumor, and initial stage. The composite numbers, while noting they include different histologies, are as follows:

Local control: 1 year 11–82%, 2 year 61–68%, 3 year 55–68.5%, and 5 year 47–82%.

Locoregional control: 1 year 66%, 2 year 58.5–76%, and 21–60% 3 year.

Disease-free survival: 1 year 51–66%, 2 year 50.6%, 3 year 55%, and 5 years 48-91%.

Overall survival: 1 year 58–88%, 2 year 20–72%, 3 year 34–70%, and 5 years 26–57%.

### 3.4. Complications (Table 3)

Complication rates ranged from 0% to 100%. The most common complications were wound infections or tissue necrosis (130; 22%), fistulae to skin (102; 18%), carotid or innominate artery blow-out (83; 14.5%), and osteoradionecrosis of facial bones (58; 10%). Acute complications accounted for 708 adverse events, of which 42% were grade I (298), 2% grade II (17), 43% grade III (302), and 13% (91) grade IV. CTCAE classifications were as follows: 19% were grade 1 (97 patients), 28% grade 2 (143), 37% grade 3 (189), 1% grade 4 (5), and 16% grade 5 (80). We determined that 66% (464) had definitive or long-term side effects, while 244 had transient short- or medium-term side effects.

**Table 3:** Complications.

Complications, *p* Value in Comparison to the Literature Without IORT		
- Wound infection/soft tissue necrosis	130	22.6%
- Oro, tracheo or pharyngo-cutaneous fistula	102	17.8%
- Carotid or innominate artery blow-out; In comparison with 4.5% in the literature: ***p* = 0.0006**	83	14.5%
- Facial osteoradionecrosis (mandible, hard palate)	58	10.1%
- Haematological	57	9.9%
- Xerostomia/mucitis	48	8.4%
- Radiation-induced neuropathy (vagus nerve, facial and hypogloss)	32	5.6%
- Trismus/loss of oral competence	15	2.6%
- Flap necrosis	14	2.4%
- Carotid occlusion with or without stroke	8	1.4%
- Hematoma	5	0.9%
- Respiratory failure	3	0.5%
- Vertebral osteoradionecrosis (one case with quadriplegia)	2	0.3%
- Meningeal fistula with or without CSF leak	2	0.3%
- Epistaxis	2	0.3%
- Brain necrosis	2	0.3%
- Hypopituitarism	2	0.3%
- Otitis/hearing loss	2	0.3%
- Late laryngeal necrosis	2	0.3%
- Skull base necrosis	1	0.2%
- Supraglottic edema	1	0.2%
- Seroma	1	0.2%
- Pleural effusion	1	0.2%
- Esophageal stenosis	1	0.2%

Short-term complications: oro-tracheal or pharyngo-cutaneous fistulas corrected by surgical revision, or by wound infections or tissue necrosis being debrided and healed; respiratory failure treated and resolved during hospitalization; cauterized epistaxis, drained hematomas, evacuated seromas, and pleural effusions treated during hospitalization. Long-term complications include all forms of osteoradionecrosis (facial, vertebral, and skull base), carotid or supraclavicular artery blow-out, neuropathies (vagus nerve, laryngeal nerve, facial nerve, and hypoglossal nerve), necrosis of reconstruction flaps necessitating another flap for reconstruction, meningeal fistulas with CSF leakage, carotid occlusion with or without stroke, brain necrosis, hypopituitarism, trismus or labial incontinence, hearing loss (sensorineural or conductive), mucositis or xerostomia, residual supraglottic edema, radiation-induced hematological disorders, esophageal stenosis, and delayed laryngeal necrosis.

## 4. Discussion

### 4.1. Indications

#### Advanced

Most authors used intraoperative radiotherapy in patients with locally advanced tumors. It is challenging to rigorously obtain the TNM stage of each patient because tumor data often come from radiotherapists’ dosimetry, which focuses more on the irradiated volume than on TNM classification. Nevertheless, most tumors were classified as stage III or IV or defined as having a volume greater than 2.5 cm^3^.

In this literature review, there are high proportions of microscopically (41%) and macroscopically involved margins (30%). Most authors believe that intraoperative radiotherapy would facilitate better local control while reducing the irradiation to remaining vital structures (such as the carotid or innominate artery, or the brain).

### 4.2. Recurrence/Prior Irradiation

The most common use of IORT is in recurrent cancer (84% of reported cases) [46]. IORT facilitates a higher overall dose of irradiation than re-irradiation alone, with the major advantages of reduced skin irradiation and the use of lead shielding to reduce the doses to critical structures such as the carotid artery.

The recurrence rates of head and neck cancers vary depending on site, stage, specific treatment, and patient-specific factors [47]. The patients undergoing IORT usually are stage III-IV and most have received prior irradiation. The lack of extensive data prevents the determination of statistics on this point.

### 4.3. Radiation Dose

Although there is no consensus on the indicated dose, the average IORT in this analysis was 10–17.5 Gy (range 1–40 Gy), after weighting the data by the number of patients reported. We saw no changes in dosage over time. The literature reports values from 10 to 30 Gy [48], depending on the type of cancer and specific treatment protocols [49]. The graphical representation of the doses administered shows some peaks resulting from certain papers that include many patients who received the same dose. However, some authors mention and detail the specific cases that led them to increase the doses. Accordingly, some doses were adjusted based on intraoperative analysis of the surgical specimen [26]. For example, Ozer et al. applied 7.5 Gy for close margins and 10 Gy for involved margins (R1 or R2) in advanced hypopharyngeal tumors. Other authors increased the dosage in previously irradiated patients; Toita et al. [13] reported increases in the average dose from 16 to 22 Gy, from 19 to 23 Gy, and from 15 to 17 Gy when there was prior irradiation, in correspondence with R2, R1, or close margins. Many authors also decreased the dose near critical structures [29,50]. For example, Mendenhall et al. reports 48 cases of nasopharyngeal tumors, in which he reduced the irradiation from 12.5 Gy to 5 Gy near the brain, 4 Gy near the optic nerve, and 8 Gy near the carotid artery.

### 4.4. Outcomes and Complications

Given the data heterogeneity in these mostly retrospective reports, it is difficult to draw definitive conclusions on IORT efficacy and outcomes. In addition, the overall management of patients has evolved over the past 40 years. The follow-up associated with patients included in reviews from the 1980s and 1990s was also less comprehensive. Some authors report an improvement in local control, with local recurrence rates decreasing from 79% to 66% at 30 months [16]. Chen et al. [27] for example reported that local control increased from 60% to 82% with IORT. However, other authors report a lack of significant improvements [35].

In this review, carotid blow-out rates were reported to be as high as 14.5%, while the literature reports a carotid blow-out rate of <3–4.5% [51]. Certainly, the higher proportion of advanced and/or previously irradiated tumors increases the likelihood of this. It has also been demonstrated that radiotherapy can elevate this rate up to 21% [52]. Whether this increased rate was due to the IORT as opposed to the fact that the tumor was advanced and recurrent cannot be ascertained in this review.

### 4.5. Limitations

IORT has several limitations, including direct costs (GBP 12,000) and the costs associated with both longer surgery and the radiation shielding in operative suites [49,53].

### 4.6. Risk of Bias Assessment

Given the retrospective and heterogeneous nature of the included studies, a formal risk of bias assessment using standardized tools (e.g., ROBINS-I or the Newcastle–Ottawa Scale) was not feasible. However, we conducted a qualitative assessment of bias. Most studies showed a high risk of selection bias due to non-randomized, monocentric designs with variable inclusion criteria. There was substantial heterogeneity in the IORT protocols (dose, modality, and timing), which contributes to a moderate to high risk of classification bias. Confounding factors—such as prior radiotherapy, tumor site, and surgical margins—were rarely controlled for statistically, leading to a high risk of confounding bias. Outcome reporting was also inconsistent, with variable definitions and follow-up durations. While some studies used established grading systems for toxicity (e.g., CTCAE), others relied on subjective or undefined criteria. These limitations were considered in our synthesis and interpretation of the results.

## 5. Conclusions

The goal of IORT is improved locoregional control of advanced and or recurrent disease, usually in cases where conventional EBRT options are limited. A higher overall dose of radiation is delivered to the tumor bed than can be delivered with EBRT alone, while seeking to spare critical structures such as the brain, optic nerve, and carotid.

Despite this theoretical advantage, and with some reports showing promise, our review highlights significant challenges associated with this technique. IORT is linked to a high rate of complications, particularly in previously irradiated tissues or when critical structures are near the irradiation field, but whether this is due to the IORT per se is not at all clear. IORT requires substantial logistical resources, including specialized equipment, extended surgical time, and multidisciplinary coordination, which limits its widespread application.

## Figures and Tables

**Table 1:** Summary of studies included.

Reference	Year Publication	Number of Patients (Sites)	Median Follow-Up (mo)	Histology ^1^	IORT Median Dose (Range), Gy	Depth; Energy; Photon/Electron;	Surgical Margins	Previous RT, %	Loco-Regional Failure, %	DFS, %	OS, %	Rate and Major Complications	Additionnal EBRT, %
**Garett et al. [8]**	1987	28 (30)	14	SCC	20 (10–25)	P	R2: 23%; R1: 27%; CM: 50%	61%	R2: 100%; R1: 275; CM: 50%	-	-	3/28: 2 V; 1 ON	-
**Garett et al. [9]**	1988	67	N/A	SCC, AD. ME, Other	1–10	P	-	-	R2: 83; R1: 16; CM: 26; R0: 0	-	-	8/67: 4 CB; 4 ON	40
**Schmitt et al. [10]**	1989	15	6–12	SCC	17.5–20	E; 6–9 MeV	-	-	R2: 100; R1: 25	-	-	CB	100
**Rate et al. [11]**	1989	47	24		20 (15–25)	P	-	100	38 (14mo)	-	55 (2y)	8/47: 1 V; 2 ON, 2 F	-
**Freeman et al. [7]**	1990	104 (109)	24	SCC, SG, S, M	15–20	E; 4 MeV	R2: 7/35; R1: 28/35	62% of SCC	R2: 43; R1: 44; CM: 30	-	-	21/104: 6% ON; 6% F; 3% CB	42
**Rate et al. [11]**	1991	47	14	SCC, AC	20 (15–25)	P	R2: 4/47; R1: 41/47	100	38.5 (2 years)	55 (29 months)	-	1 CB; 2 ON; 3 F	-
**Braun et al. [12]**	1991	1			20	P	-	100		-	-		-
**Freeman et al. [12]**	1991	25	12		15–20	E; 4 MeV	-	yes without precision	33%	-	-	2 ON	-
**Toita et al. [13]**	1994	25 (30)	19 (3–59)	SCC	R2: 22 (15–30) if not post RT, 16 (15–20) if post RT; R1: 23 (20–30) if not post RT, 19 (15–25) if post RT; CM: 15 (15) if post RT, 17 (10–20) if not post RT	E; 6–18 MeV (median 9 MeV)	R2: 9.3%; R1: 33.3%	-	-	- R2: 100; - R1: 55.5; - R0: 19.9; - Overall: 22	-R2: 0; -R1: 54.5; -CM: 81.8. -Overall: 54.1 (2y)	5/23: 3 CB; 6 ON	66
**Wolf et al. [14]**	1995	5	20–48	T	4–6	P	1 R2; 2 R1; 2 CM	-	R2: 50; R1: 0	-		1 F	100
**Freeman et al. [15]**	1995	75	24	SCC; M; ME; AC	17.5 (10–25)	E: 5–11 MeV	-	61	overall: 32 (2y); R2 75; R1: 27; CM: 24	-	45 (2y)	19/75: 1 CB; 4 V; 4 F; 2 N; x ON	33
**Nag et al., [16]**	1995	29	21		7.5–15	P	-	-	overall 11; 67 if only IORT, (21mo)	-	overall 72, 100 if IORT + EBRT, 17 if only IORT,	CSF leak	58
**Ling et al. [17]**	1996	30	30	SCC, ME, AC, AD, A	15	E: 6–9 MeV	-		overall: 40 (3y)	-	70 (3y)		-
**Coleman et al. [18]**	1997	44 (46)	24	SCC, ME, AD, AC, PD; A, chrondrom	14–18	E: 2.5–9.5 MeV; 2.5–9.5 mm	-	39	39 (20mo)	61.7 (2y)	66 (2y)	8/44: 3W; 1CB; 1 ON, 3N	37
**Spaeth et al. [19]**	1997	95 (120)	11	SCC, AD, M, S, ME, A, LE, H, N-HL	20 (10–40)	P	-	95	89 (11mo)	-	20 (2y)	11/120: 8W; 3F	
**Schmitt et al. [20]**	1997	43	24	SCC	20–25	E: 6 MeV; 1–2.7 cm	_	_	overall 66 (3y); 51 (5y)	-	60 (5y)	3FN; 1LN; 1LS	100
**Nilles-Schendera et al. [21]**	1997	42	_	SCC	12–15	P	_	_	8/42 (6y)	-	-	0	66
**Martinez-Monge et al. [22]**	1997	31	24	SCC mostly	10–15	P	-	47	67 (2y)	-	20 (2y)	7/31: 5 F; 1 FN	-
**Nag et al. [16]**	1998	38 (40)	30	SCC, ADK, LC	15 (15–20)	P	-	100	79 (30mo)	-	21 (2y)	6/38: 1W; 2 V; 2 F; 1 CO	-
**Schendera et al. [21]**	1998	58	60		12–25	P	-	overall: 19; Gp III: 100	-	-	Gp I: 32 (5y); Gp II: 90 (5y); Gp III: 14 (3mo)	-	-
**Schleicher et al. [23]**	2001	84 (113)	6.8	SCC, T, SG	20 (10–20)	P	-	100	R2: 76; R1: 58; R0: 50	-	37 (1y)	21/84: 3.5%F, 2%N; 9%W	-
**Schuller et al. [24]**	2002	43	14.6	SCC	7.5–10	6 MeV, P and E, 5 mm	-	-	overall 7	-		6 F, 1FN, 1 N	100
**Pinheiro et al. [25]**	2003	44 (50)	75.6 for survivors	SCC, non SCC	12.5–22.5	P	-	-	54 SCC, 48 non-SCC (2y)	-	32 SCC; 50 non-SCC (2y)	23/44: 8 W; 2 CB; 3 F; 5 N	-
**Ozer et al. [26]**	2006	32	89 (3.4–140)	SCC	7.5–10	6 MeV, P and E, 5 mm	-	-	9 (5y)	-	Overall 56 (5y)	-	yes, not specified
**Chen et al. [27]**	2007	137 (191)	41 (3–122)	SCC, AC, ME, AD, S, M; AcC	15 (10–18)	E	-	-	in field: 38; locoregional: 49 (3y)	-	overall 36 (44 if primary recurrence, 19 if neck recurrence)	4/137: 2 F, 1 Ne	26
**Schuller et al. [28]**	2007	123	60	SCC	7.5–10	E	-	-		73 (5y)	57 (5y)	29/123: 4W; 19F; 1 FN; 2ON	yes, not specified
**Mendenhall et al. [29]**	2008	86	Nasopharynx 60; H&N: 8-39	NP, SCC, AD, LC	4–12.5	P	-	-	Nasopharynx 53 (5y) H&N: 87 in field; 96 locoregional (2y)	-	Nasopharynx: 47 (5y); H&N: 8 (2y)	2 F; 1 CB; 7 BN; 2 Hy; 6 N; 2ON	
**Marucci et al. [30]**	2008	25	10	SCC, DC	12	7–9 MeV; P; 4–8 cm	-	-	41.5 (2y)	50.6 (2y)	64.5 (2y)	6/25: 1W; 1 ON; 3 F; 1 FN	
**Chen et al. [31]**	2008	37	44.4	SG	15 (15–18)	P	-	-	18 (w) vs. 40 (w/o IORT), *p* = 0.001	-	54 (3y); 34 (5y)	4/37: 2W, 1N	15
**Most et al. [32]**	2008	21		SCC, AD, ME, S	10–15	P	-	-		-		1 FN; 1 CB; 1F	28
**Kopp et al. [33]**	2009	47		SCC, AD, undifferentiated, AC, EB, NC, ME, other	10	P	-	-	-	-	41 if primary, 33 if recurrence (5.7y)	-	70
**Perry et al. [34]**	2010	34			15	P	-	-	34 (1y); 44 (2y)	-	27 (1y); 45 (2y)	-	
**Joos et al. [35]**	2010	60 (30 w IORT, 30 w/o IORT)	2y	SCC mostly	15 (10–18)	-	-	-	-	-	IORT group: 23.3 if R0, 50 if R1, 26.6 if R2 (2y). No difference w or w/o IORT	-	-
**Rutkowski et al. [36]**	2010	16	30 (0–66)	SCC	5–7.5Gy	P; 20 KeV	-	0	Local: 0; Locoregional: 18	-	81	3/16: Other	100
**Harrison et al. [37]**	2011	90	11	SCC, SG, S, Skin, EB	12 (10–17.5)	P; 1 cm	51% positive margins	-	in field: 35; out of field: 22 (1y)	51 (1y)	66 (1y)	1 ON, 1 CB	100
**Zeidan et al. [38]**	2011	46	67.2	SG	15–20	P	-	-	-	51.9 (5y)	59 (3y); 48 (5y)	4% ON, 4% FN, 1% N	57
**Zieden et al. [39]**	2012	96	67.2	ME, SCC, AC, AD	15–20	P	-	35.5	-	82 (1y); 68.5 (3y); 65.2 (5y)	88.4 (1y), 66.1 (3y); 56.2 (5y)	26/96: 2 W; 7V; 4 F; 4 ON; 1 N	-
**Zieden et al. [38]**	2012	231			15–20	P	-	-	-	66 (1y); 55 (3y), 49 (5y)	58 (1y);34 (3y); 26 (5y)	20/231: 23V; 8ON; 20 F; 2FN	-
**Scala et al. [40]**	2013	76	12–60	SCC, AD, other	10–17.5	P	-	-	44 (1y); 48 (2y)	-	64 (1y); 42 (2y)	4% CB, 1% N	N/A
**Majercakova et al. [41]**	2014	9		SCC	8 (8–12)	P; 5 mm	-	-	-	85% (w) vs. 66% (w/o).		none	-
**Enami et al. [5]**	2016	12	5–18	parotid and other	6 (5–14)	P; 5 mm	CM or clinical impression of CM	42	5 mm; P; 50 keV	-	-	none	50
**Cristalli et al. [42]**	2016	13	33		12	P; 12–17 mm	No R2	-	-	68 (5y)	-	1 FN, 1 F	100
**Emami et al. [5]**	2017	22	16 (3–33)	SCC, SG	12–14	P; 4–6 cm	-	-	13 (1.5)	-		2 N	45
**Yi et al. [43]**	2017	9	14–22 (2 lost)	T	3–8	P; 50 KeV	-	-	-	89 (5mo)	-	1 F, 1 W	11
**Moubayed et al. [44]**	2017	1	-	SCC	6	P	-	-	-	-	-	1 F	-
**Wald et al. [45]**	2019	61	15.9 (4.9–74.4)	SCC	12.5 (10–17.5)	P	46% R3, 44% R-, 10% unkmown	95 (1–277 mo)	41 (1y)	39 (1y)	62 (1y); 42 (2y)	1 CB	38

^1^ SCC: Squamous Cell Carcinoma; AD: adenocarcinoma; ME: Mucoepidermoid Carcinoma; SG: Salivary Gland Tumor; M: Melanoma; T: Thyroid Tumour; AC: Adenoid Cystic Carcinoma; A: Anaplasic Carcinoma; PD: Poorly Differentiated Tumour; S: Sarcoma; LE: Lymphoepithelial; H: Histiocytome; N-HL: Non-Hodgkin Lymphoma; LC: Large Cell Tumour; AcC: Acinic Cell Carcinoma; NP: Nasopharyngeal Carcinoma; DC: Ductal Carcinoma; EB: Esthesioneuroblastoma; NE: Neuroendocrine Carcinoma. ON: Osteoradionecrosis; CB: Carotid Blowout; W: Wound dehiscence or infection; N: Radioneuropathy; FN: Flap Necrosis; F: Fistula; V: Vascular complication; CO: Carotid Occlusion; LN: Laryngeal Necrosis; LS: Laryngeal Stenosis; BN: Brain necrosis; Hy: Hypopituitarism.

## Data Availability

No new data were created or analyzed in this study. Data sharing is not applicable to this article as it is based on a review of previously published studies.

## Supplementary Materials

The following supporting information can be downloaded at: https://www.mdpi.com/article/10.3390/cancers17132124/s1.
