# Supplementary material for: Systematic Review of Intraoperative Radiotherapy (IORT) in Head and Neck Oncology: Past, Present, and Future Perspectives
Source: Cancers (Basel). 2025 Jun 24;17(13):2124. doi: 10.3390/cancers17132124 (PMC12249129; doi:10.3390/cancers17132124)
Supplement: Supplementary file 1 [file cancers-17-02124-s001.zip › IORT Protocole.pdf]

PRISMA-P (Preferred Reporting Items for Systematic review and Meta-Analysis Protocols) 2020 checklist: recommended items to address in a systematic review protocol*

Section and topic	Item No	Checklist item
**ADMINISTRATIVE INFORMATION**		
Title:		
Identification	1a	Intra-Operative Radiotheray in Head and Neck Surgery, a systematic review
Update	1b	None
Registration	2	None
Authors:		
Contact	3a	Pincet Laurence – Head and Neck Department – CHUV – Rue du Bugnon 46 – 1011 Lausanne<br>Fanchette Aurelie– Head and Neck Department – CHUV – Rue du Bugnon 46 – 1011 Lausanne<br>Simon Christian – Head and Neck Department – CHUV – Rue du Bugnon 46 – 1011 Lausanne<br>Lambercy Karma – Head and Neck Department – CHUV – Rue du Bugnon 46 – 1011 Lausanne
Contributions	3b	Dr Pincet and Dr Fanchette selected the articles. In case of hesitation, Pr Simon participated to the final decision. Dr Pincet and Dr Fanchette wrote the article. Dr Lambercy and Pr Simon supervised and validated the last version of the manuscript.
Amendments	4	This review completes the already existing literature review*, by updating the data, and with a clearer protocol.<br>* : Kyrgias, George; Hajiioannou, Jiannis; Tolia, Maria; Kouloulias, Vassilios; Lachanas, Vasileios; Skoulakis, Charalambos; Skarlatos, Ioannis; Rapidis, Alexandros; Bizakis, Ioannis (2016). Intraoperative radiation therapy (IORT) in head and neck cancer. Medicine, 95(50), e5035–. doi:10.1097/MD.0000000000005035
Support:		
Sources	5a	None
Sponsor	5b	None
Role of sponsor or funder	5c	N/A
**INTRODUCTION**		
Rationale	6	Intraoperative radiotherapy has been used in ENT for about 30 years in many centers. It allows a high dose of radiation to be delivered as close as possible to the tumor bed, while sparing nearby healthy structures. However, recommendations for the management of patients with head and neck cancer include conventional external radiotherapy, and intraoperative radiotherapy is hardly finding its place in oncologic management schemes.
Objectives	7	We would like to review the use and outcomes of IORT in head and neck surgery to update the state of the art in this area. We are interested in all patients operated on for head and neck cancer, who have received one or more sessions of intraoperative radiotherapy. We will report a synthesis of the survival of these patients, the reported side effects, the material constraints that such a treatment generates.

**METHODS**		
Eligibility criteria	8	The search terms includes :<br><br>- intraoperative radiotherapy<br>- Intraoperative Radiation Therapy<br>- IORT<br>- IOERT,<br>- intraoperative RT,<br>- intraoperative radiation<br><br>AND<br><br>- cancer of head and neck<br>- head and neck neoplasms<br>- head and neck cancer<br><br>***within all the sub-groups :***<br><br>- Esophageal Neoplasms<br>- Esophageal Squamous Cell Carcinoma<br>- Facial Neoplasms<br>- Eyelid Neoplasms<br>- Mouth Neoplasms<br>- Gingival Neoplasms<br>- Leukoplakia, Oral +Lip Neoplasms<br>- Palatal Neoplasms<br>- Salivary Gland Neoplasms +Tongue Neoplasms<br>- Otorhinolaryngologic Neoplasms<br>- Ear Neoplasms<br>- Laryngeal Neoplasms<br>- Nose Neoplasms +Pharyngeal Neoplasms +Parathyroid Neoplasms<br>- Squamous Cell Carcinoma of Head and Neck<br>- Thyroid Neoplasms<br>- Thyroid Cancer, Papillary<br>- Thyroid Nodule<br><br>Articles published in any language other than English, are excluded.<br><br>In terms of study design, selection is restricted to systematic reviews, meta-analyses, clinical trials, cohort studies, case–control

		studies, and case series;<br><br>In terms of sample size, there is no restriction in number of patients who received treatment with IORT;<br><br>In terms of the target disease, adult patients with head and neck cancer of any histology and extension were eligible;<br><br>In terms of survival, we included studies that assessed survival with a mean or median follow-up of 3 months or longer.<br><br>In terms of publication status, we included only published studies, with no anterior limit date of publication, until now.
Information sources	9	Medline, Scopus, Ovid, Cochrane, Embase, and ISI Web of Science
Search strategy	10	Present draft of search strategy to be used for at least one electronic database, including planned limits, such that it could be repeated
Study records:		
Data management	11a	We will use EndNote® and Ryyan® to select the articles
Selection process	11b	We will review the selected articles with two independent reviewers (screening, eligibility and inclusion in literature review)
Data collection process	11c	Datas will be resumed in an Excel table.
Data items	12	References, Years of trial, Number of patients, Median follow-up, Histological findings, Dose of IORT, Surgical margins, Previous radiotherapy, Adjuvant treatments, Local failures, Loco-regional failures, Cumulative failure, Disease-Free Survival, Overall survival, Surgical complication, IORT adverse events, Authors' indication for IORT.
Outcomes and prioritization	13	Locoregional control
Risk of bias in individual studies	14	Describe anticipated methods for assessing risk of bias of individual studies, including whether this will be done at the outcome or study level, or both; state how this information will be used in data synthesis
Data synthesis	15a	We will do an observational study, no meta-analysis. We don't plan of doing statistics.
Meta-bias(es)	16	N/A
Confidence in cumulative evidence	17	N/A

*** It is strongly recommended that this checklist be read in conjunction with the PRISMA-P Explanation and Elaboration (cite when available) for important clarification on the items. Amendments to a review protocol should be tracked and dated. The copyright for PRISMA-P (including checklist) is held by the PRISMA-P Group and is distributed under a Creative Commons Attribution Licence 4.0.**

From: Shamseer L, Moher D, Clarke M, Ghersi D, Liberati A, Petticrew M, Shekelle P, Stewart L, PRISMA-P Group. Preferred reporting items for systematic review and meta-analysis protocols (PRISMA-P) 2015: elaboration and explanation. BMJ. 2015 Jan 2;349(jan02 1):g7647.
